# Remembering Eliahu de Luna Montalto (1567–1616)

**DOI:** 10.5041/RMMJ.10285

**Published:** 2017-01-30

**Authors:** George M. Weisz, Donatella Lippi

**Affiliations:** 1 School of Humanities (Program in History of Medicine), University of New South Wales, Sydney, Australia; 2 School of Humanities, University of New England, Armidale, New South Wales, Australia; 3 Center for Medical Humanities, Department of Experimental and Clinical Medicine, University of Florence, Italy

**Keywords:** Eliahu de Luna Montalto, history of medicine, Inquisition and doctors, Jewish doctors

## Abstract

Born in Portugal and the son of Marranos (Christianized Jews from Spain), Eliahu de Luna Montalto lived during a particularly harsh period for the Jewish people. Throughout Europe, the situation for Jews was unfavorable; laws had been passed forbidding them to live in England for the past 300 years, and for the past 200 years in France. Additionally, in France, while Jews were permitted to study at some universities, the practice of medicine was forbidden to them. It is within this context that Eliahu de Luna Montalto, who had returned to his original faith (Judaism), was recruited to the French court. This paper pays tribute to Montalto’s life and medical practice—so exemplary that the Queen of France would ask Montalto to serve at the court and receive Papal permission for Montalto openly to observe his faith as a Jew, this despite the objections of the King of France.

## BACKGROUND

During mediaeval times, Catholic theologians proposed that rather than exterminate the Jews, they should be kept in permanent wandering for their sins. These rules were more harshly enforced upon the Jewish community during the Renaissance, with the introduction in Portugal in 1536 of institutional persecution and conversion; Jews who converted were referred to as *Conversos* or *Marranos* (“pig” in Spanish).^1^ Around the same time, the Pope also issued a harsh anti-Jewish Bull that was re-enforced by two of his successors.

Reflecting the times in which he lived, the King of France, Henry IV, did not allow Jewish physicians to work in Paris. Interestingly, he had left his Huguenot upbringing and converted to Catholicism in order to be crowned.

Despite this atmosphere that pervaded Europe, Jewish physicians were kept in high esteem. Historically, the hypocrisy of monarchs and pontiffs is well known. While officially they persecuted the Jews, in private life, once a monarch was in need of a physician, the physician’s faith was ignored.^2^ It is somewhat anecdotal that King François I asked for a Jewish physician from Spain and then rejected his services upon learning that the doctor was a convert to Judaism and not a “real” Jew.^3^,^4^

One of the most typical examples of monarchial hypocrisy relates to Dr Elijah de Luna Montalto, who made significant contributions to medicine, morality, and theology.^5^–^14^

## LIFE AND TIMES

Montalto was born in Castelo Franco, Portugal in 1567 to Marrano parents—both of whom came from a legacy of medical practice. Montalto grew up as a Christian and only became aware of his Jewish roots in adulthood. His birth name was Felipe. Montalto studied medicine in Salamanca, Spain, one of the oldest Universities in Europe and returned to Portugal to practice for one year, but his family was eventually obliged to leave.^3^–^13^ Montalto traveled through Southern France, to Amsterdam and enjoyed a brief respite in Paris. His name must have gone ahead of him, since he was summoned by the Queen, Maria de Medici of the house of Bourbon (also known as Marie Medici/s), to help her childhood friend and confidante, Léonora Galigai-Concini, despite the opposition of the Queen’s husband, King Henry IV.^6^,^7^

Montalto gradually became more aware of his Jewish roots and changed his name from Felipe (meaning “lover of horses”) to Philotheo (“lover of God”). He moved to Livorno, where the pragmatic Grand Duke of Tuscany, Ferdinando I de Medici, allowed for the settlement of a very productive Jewish community. Montalto eventually ended up in Florence and became court physician to the Medici family.^11^,^14^ In this relatively liberal atmosphere he studied Judaism under Daniel Franco, who would eventually be burned at the stake for his Jewish faith after returning to Portugal.^9^,^10^

After some two years, Montalto secretly abandoned his high position and significant revenue in Florence to relocate in the Ghetto of Venice, where some 6,000 Jews lived in poverty and were in great need of medical assistance.^8^,^9^,^14^,^16^

Montalto lived and worked in the ghetto for four years. During this time, the Serenissima Republic was outside of the Vatican’s control and was therefore tolerant of the Jews. Nevertheless, Jews lived on an island with one bridge connecting them to the rest of the city. Only physicians-on-call were allowed to leave the island at night, provided the guards were paid a certain fee.^16^ In 1612, at the request of the French monarch, and only after certain preconditions had been met, Montalto moved to Paris and again served as the court physician, primarily to Maria de Medici.

## MONTALTO’S MEDICAL PRACTICE

Montalto promoted the four temperaments of humorism first proposed by Hippocrates and Galen; later on he also promoted astrology. Based on his writings, Montalto’s approach seemed to be more psychological than medical; he must have been successful, as his services remained in demand throughout his life.

Only one full case report related to his treatment of a patient is recorded, that of the French Queen’s controversial confidante, Léonora Galigai-Concini. Montalto diagnosed her with “Bulbus hystericus” and recommended a conservative, non-invasive treatment approach comprised of a change in diet, fresh air, daily walks, minor exercises, and sexual abstention for six weeks. His treatment of her apparently helped.^7^,^12^ She herself testified to that in 1606.^14^^(p54),^^17^ Interestingly, Léonora would eventually be beheaded for being a Jewish witch in 1617 (one year after Montalto’s death).^18^

Montalto would later write a treatise on love-sickness and list the treatments he had recommended to be used for Léonora’s case.^14^ In modern times, Léonora’s illness might have been interpreted as a psychosomatic disease. History provides ample evidence of her possible psychological problems. Hers was a marriage of convenience, and only after the wedding did she learn that her husband had a different sexual orientation. She was also deeply involved in mysticism.^18^ Hence, Montalto may well have diagnosed what is now understood to be a psychosomatic disease; his treatment could be viewed as an early form of psychotherapy, although such a diagnosis and treatment had not yet been defined.^6^

Montalto is particularly recognized for his contributions in the form of medical publications, particularly two books with extant copies available only in major European libraries. His books were reviewed in 1929^8^ and the 1930s;^2^,^6^ some commentary on his work, with inconsistent data, was published in 1980s;^3^,^4^,^9^,^11^,^12^ and a more recent psychiatric review was published in 2003.^5^

### Optica

Montalto’s first book was *Optica: on vision, on the visual organs and theory of vision*, written in Latin and published in 1606.^15^ The contents of the book, in addition to the preface, provide ample evidence of Montalto’s medical expertise: he writes that he had been offered a position in the medical faculty of the University of Rome, a position he declined due to fear of compromising his religious convictions.^13^,^14^

**Figure 1:**
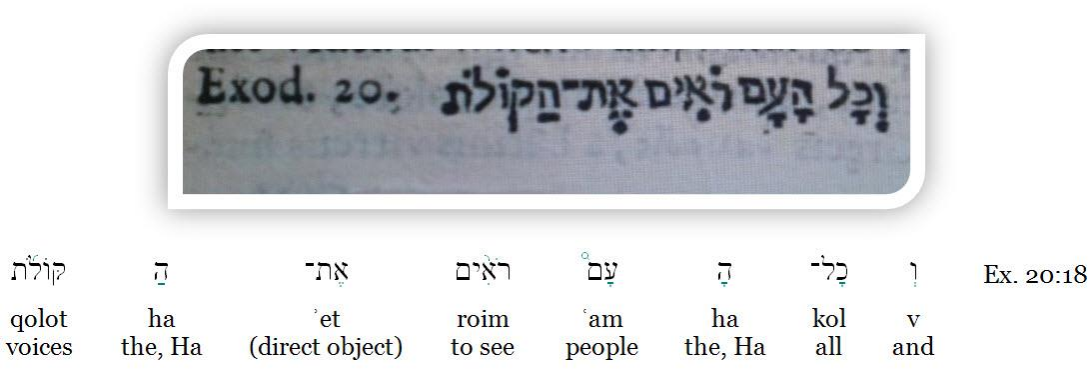
Quote from Montalto’s *Optica*. For the purposes of clarity, a parsing of the verse “*v’kol ha-am roim et haqolot*” (“... and all the people saw the voices”) is provided beneath the picture.

The book includes chapters discussing the superiority of vision to all other senses, a description of the structure and nature of the eye (with no autopsy details), and he proposes that vision is actually generated in the brain and not in the eye; he also states that vision results from rays emitted from the eye through images received in the eye. In support of his theories, Montalto quoted Exodus 20:18, which literally interpreted reads: “*All the people saw the voices*” (Figure 1). Hence, he believed that sound was transformed into images and perceived in the brain.

Another chapter in *Optica* dealt specifically with the function, form, and position of the lens, and yet another one with eye color. The book was considered to be psychologically descriptive rather than a study of vision (optics). The book was widely disseminated, and a second edition was reprinted in 1613. The book must be considered as scientifically precocious for 1604.

### Archipathologia

Montalto’s second major work was *Archipathologia*, dated 1614, a psychological treatise on mental and neurological disorders. This volume included chapters on pain, headaches, delirium (brain fever), melancholy, coma, insomnia, lethargy, incomplete unconsciousness (*Coros*), catalepsy, vertigo, night-mare, epilepsy, and apoplexy. A total of 18 “tractates,” over 817 pages, covered all mental disturbances known at that time.^18^ In each chapter, Montalto offered a Hippocratic explanation, described the symptomatology, established the diagnosis, assumed the prognosis, and, finally, gave a therapeutic suggestion. Most remarkable were the pharmaceuticals offered for each condition.^6^,^18^

## THE IMPACT OF MONTALTO’S MORALITY ON HIS MEDICAL PRACTICE

The moral and ethical practices of Montalto were a finely balanced mixture of his religious beliefs and medical professionalism. At a time when anti-Semitism was tempered by expediency, Montalto not only wrote a commentary, but participated in debate with a Dominican theologian on the differences between the two testaments. This disputation is outside the scope of this article.^13^ It is, however, noteworthy that Montalto was the first known Sephardi anti-Christian polemicist and that his public stance did not impede his medical practice.^19^ This too is mute evidence to the respect with which his medical practice was received.

As discussed above, Montalto reconverted to Judaism in adulthood. Unlike many of his compatriots, he did not hide his faith. He tried to convince his brother-in-law to re-embrace Judaism; he left his high position in Florence, and would go on to refuse several university professorships from fear that it might “interfere with his religious practices.”^6^,^14^ He opted instead to serve as a much-needed physician in the Venetian ghetto.

Thus, it was no small thing for him to leave the ghetto and return to Paris. In 1611, Montalto was summoned by the French Queen. His reply was that it would be an honor to accept, but that he needed assurances that he would “be able to freely practice Judaism, not be called on the Sabbath and not be offered money on the Sabbath.”^7^–^10^

Despite the opposition of the King, the Queen used the connection with her uncle, the Grand Duke Medici, to obtain a Papal dispensation for an “infidel” to practice in the French capital. Montalto moved to Paris in 1612 with his wife and two children and an accompanying historian.^11^,^12^

His patients were the children of the monarchs (including the future Louis XIII) and the court aristocrats. Montalto’s life in Paris was professionally intense. Nevertheless, he was allowed openly to practice Judaism, although at least a dozen other co-religionists remained hidden. He protected the small secret Jewish community in Paris and one in Lyon. Once they were discovered, Montalto managed to obtain reversal of the King’s order to expel them from the land.

## MONTALTO’S DEATH AND BURIAL

The French Court was full of intrigues, rivalries, and violence, including the murder of King Henry IV in 1610. The King’s court frequently traveled throughout France. In May 1616, while in Tours, Montalto became ill and died within 10 days—reportedly from the plague.

**Figure 2:**
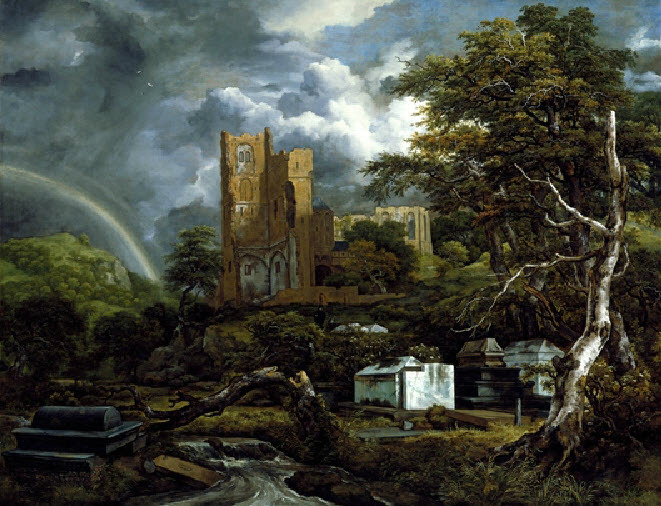
Jacob Isaaksz van Ruisdael, *The Jewish Cemetery*, 1654 or 1655, Oil on Canvas. Detroit Institute of Arts, 26.3. Used with permission of the Detroit Institute of Arts.

It is perhaps fortuitous that he died when he did—otherwise he might have suffered the same fate as his patient Léonora Galigai-Concini.^17^ However, Montalto escaped the more severe court intrigues that were to come and was treated by the Queen with great honor—even after his death. As there was no Jewish cemetery in France, at the Queen’s demand Montalto’s body was embalmed and sent to Amsterdam where he was interred in the Ouderkerk Cemetery. His tomb was engraved in both Latin and Hebrew with the words, “Eliahu Montalto physician to the Queen of France.” The grandeur of his tomb was immortalized by the famous Dutch painter Jacob Isaaksz van Ruisdael in two landscapes, which are today on display in museums in Detroit, Michigan, USA and Dresden, Germany (Figure 2).^20^

## CONCLUSION

In our own age, when men and women worldwide are being encouraged and even forced to separate the practice of their faith from their profession, Eliahu Montalto is a refreshing example of a man of conviction. His contribution to medicine was advanced for his time despite some of the spurious theories he entertained. Had he chosen to remain a Marrano and live his life as a Christian, history would undoubtedly have taken greater note of his accomplishments. His two books and multiple tractates continue to be perused in order better to understand the time in which he worked and the thinking process and understanding of his peers.

The hypocrisy of Montalto’s times are a stark warning to us today. Medicine must not be compromised for the sake of faith; but faith likewise must not be compromised for the sake of political correctness. Many physicians in history have been equally great men of faith. While our goal is to bring to light the contributions of overlooked Jewish doctors and scientists, men and women of faith have contributed throughout history to medicine and medical advancement. Their achievements and example, like those of Montalto, must not be ignored.
